# A Novel Model of Human Skin Pressure Ulcers in Mice

**DOI:** 10.1371/journal.pone.0109003

**Published:** 2014-10-13

**Authors:** Andrés A. Maldonado, Lara Cristóbal, Javier Martín-López, Mar Mallén, Natalio García-Honduvilla, Julia Buján

**Affiliations:** 1 Department of Plastic and Reconstructive Surgery and Burn Unit, University Hospital of Getafe, Madrid, Spain; 2 Department of Pathology, University Hospital of Puerta de Hierro, Madrid, Spain; 3 Department of Genetics, University Hospital Central de la Defensa, Madrid, Spain; 4 Department of Medical Specialties, Faculty of Medicine, University of Alcalá, Networking Research Centre on Bioengineering, Biomaterials and Nanomedicine (CIBER-BBN), Madrid, Spain; Tel Aviv University, Israel

## Abstract

**Introduction:**

Pressure ulcers are a prevalent health problem in today's society. The shortage of suitable animal models limits our understanding and our ability to develop new therapies. This study aims to report on the development of a novel and reproducible human skin pressure ulcer model in mice.

**Material and Methods:**

Male non-obese, diabetic, severe combined immunodeficiency mice (n = 22) were engrafted with human skin. A full-thickness skin graft was placed onto 4×3 cm wounds created on the dorsal skin of the mice. Two groups with permanent grafts were studied after 60 days. The control group (n = 6) was focused on the process of engraftment. Evaluations were conducted with photographic assessment, histological analysis and fluorescence in situ hybridization (FISH) techniques. The pressure ulcer group (n = 12) was created using a compression device. A pressure of 150 mmHg for 8 h, with a total of three cycles of compression-release was exerted. Evaluations were conducted with photographic assessment and histological analysis.

**Results:**

Skin grafts in the control group took successfully, as shown by visual assessment, FISH techniques and histological analysis. Pressure ulcers in the second group showed full-thickness skin loss with damage and necrosis of all the epidermal and dermal layers (ulcer stage III) in all cases. Complete repair occurred after 40 days.

**Conclusions:**

An inexpensive, reproducible human skin pressure ulcer model has been developed. This novel model will facilitate the development of new clinically relevant therapeutic strategies that can be tested directly on human skin.

## Introduction

Pressure ulcers (PU) are a high-prevalence problem in our society. It is estimated that 1.3 million to 3 million adults have a PU, with an estimated cost of $500 to $40,000 to heal each ulcer. The incidence varies greatly by clinical setting; in the hospital, for example, the incidence is estimated to be 0.4% to 38.0% [1].

Although conservative management is conducted in clinical practice (e.g., postural changes, dressing care), there is great disparity in the approach and management of these patients [2], [3]. According to the American and European Pressure Ulcer Advisory Panel guidelines, nutrition is an important aspect of a comprehensive care plan for prevention and treatment of pressure ulcers (although limited evidence-based research is available) [4]. Moreover, according to Thomas [5], prescriptions should be individually tailored to persons with pressure ulcers with regard to both macro- and micronutrients. Surgical treatment is used in only a small numbers of patients. However Larson and others advocate good results with a surgical approach (without consideration of nutritional status or osteomyelitis) [6], while other authors have reported a high recurrence rate with this method [7], [8].

Approaches incorporating cellular therapy and growth factors are thought to be on the horizon. The combined clinical evidence on platelet-derived growth factor (PDGF) suggests that PDGF-BB may improve healing of pressure ulcers. However, the evidence is not sufficient to recommend this treatment for routine use [9]. According to Akita et al. [10] adipose-derived stem cells can promote human dermal fibroblast proliferation by directly contacting cells and via paracrine activation in the re-epithelialization phase of wound healing. Moreover, skin substitutes were made by employing advanced tissue-engineering approaches and have been used for clinical applications, promoting the healing of acute and chronic wounds [11]. For example, bilayered bioengineered human skin equivalent (Apligraf, Novartis) has been shown to be efficacious in a case study of patients with heel PUs (level IV evidence) [12]. Additionally, growth factors could be another alternative to stem cells. According to Yang et al. [13], the expression of VEGF and bFGF in PU tissue is decreased. This leads to a reduction in angiogenesis, which may be a crucial factor in the formation of PUs.

Regarding the etiology of PUs, external pressure is viewed as the main factor. Other patient-specific factors leading to derangement in tissue perfusion may account for an observed development of a pressure ulcer [14]. It is well known that ischemia–reperfusion injury contributes to the pathophysiology of PUs more significantly than a single, prolonged ischemic insult [15]. The animal models described in the literature employ a variety of devices to apply localized pressure on the back of mouse skin, and many of these models use external magnets; this technique is based on the repetition of ischemia-reperfusion cycles [16]–[19]. However, all of these models are based on mouse skin, which could be a potential limitation to studying the effect of human stem cells or growth factors in the PU environment.

From our point of view, the shortage of suitable animal models together with the ethical and practical considerations for humans limits our understanding of PUs and the development of new therapies. This study aims to report a novel and reproducible PU model of human skin graft. Cell therapy, growth factors and other techniques could be applied directly to human skin instead of mouse skin.

## Material and Methods

### Animals

Three-week-old, male, non-obese diabetic/severe combined immunodeficiency (NOD.CB17-Prkdscid/NCrHsd) mice (n = 22) (Harlan Laboratories S.r.l. Barcelona, Spain) were used in this study. All mice were caged under standard light and temperature conditions with free access to food and water throughout the study. All experimental procedures were made to minimize suffering and they were approved by the local committee for animal welfare and were conducted in accordance with the European Community Council Directive (86/609/EEC). The ethical Committee at University of Alcalá (Madrid, Spain) approved this research.

### Human skin grafts

Mice were engrafted under general anesthesia (Ohmeda, BOC Health Care) with female human skin. All human skin came from abdominoplasty or breast reduction procedures. Ethics committee from University of Alcalá (Madrid, Spain) was approved and written informed consent from all patients was obtained. Full-thickness skin grafts (FTSGs) were placed onto a 4×3 cm wound created with scalpel on the dorsal skin of the animals. Mice skin was incised down to the muscle and removed, exposing muscular layer. FTSGs were sutured in place with 4/0 nylon. Postoperative analgesia (meloxicam and buprenorphine) was provided for 3 days and the dressing was tied on for the first 5 days. Special care was taken with sterilization and postoperative animal handling due to the immunodeficient status of the mice. A total of 4 mice died in the immediate postoperative period.

Mice were classified into two groups after 60 days with permanent human skin grafts. In the control group (n = 6), mice were sacrificed after 190 days and the FTSG was removed to study the engraftment process.

### Pressure ulcers

**Figure 1:**
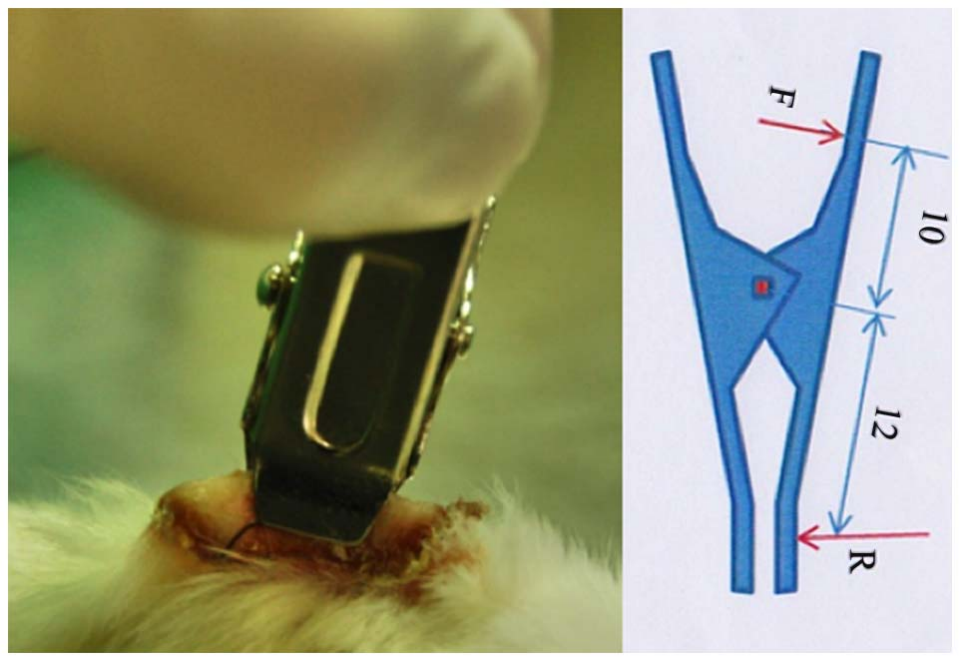
Compression device on the human full-thickness skin graft delivering a pressure of 150 mmHg. A, three cycles of compression (8 h of clamping after 16 h of no compression) were delivered to the human skin graft to generate the pressure ulcer. B, schematic representation of the compression device. F =  force generated by the spring. R =  force generated by the compression device.

In the PU group (n = 12), mice were placed in a compression device (Fig. 1A) following the method described by Stadler et al. [17] and modified by our group in terms of the device and the timing. A modified pressure device (7 mm×5 mm) that delivered a pressure of 150 mmHg to the human FTSG was used. The exerted pressure was measured with a dynamometer (Fig. 1B). Three cycles of compression-release (8 hours of clamping after 16 hours of no compression) were applied to the human FTSG. This group was subdivided into two groups. From the first one (n = 6), biopsies were taken at 5, 25, 45 and 130 days post-cycle. From the second subgroup (n = 6), animals were used to assess PU evolution by photographic analysis. All animals were sacrificed after 190 days.

### Macroscopic analysis

At the macroscopic level, the behavior of the graft was evaluated every 15 days for 60 days in the control group. In the PU group, the PU was evaluated every 7 days for 130 days after compression cycles. The evolution of the graft was analyzed morphometrically (ImageJ for Windows XP NIH Image) using photographs from days 0 to 190. These measurements were taken by 2 independent researchers who were blinded to the treatment group. The values are expressed as the means ± standard deviation.

### Microscopic evaluation

At the end of the experiments, tissue specimens were collected for different studies and placed in 10% buffered formaldehyde, Bouin and Carnoy. Then the samples were dehydrated and embedded in paraffin. Tissue sections (5 µm-thick) passing through the center plane of each wound were stained with hematoxylin-eosin and Masson's trichrome for morphological assessment. Cytogenetic analysis using fluorescence in situ hybridization (FISH) for the X and Y chromosomes (sonde XA X/Y, D-5608-100-OG, MetaSystems GmbH) was also performed.

### Statistical analysis

Areas of the FTSG were compared among treatment groups by ANOVA followed by the Mann-Whitney U test. Differences with p<0.05 were considered statistically significant.

## Results

### Human skin grafts

**Figure 2:**
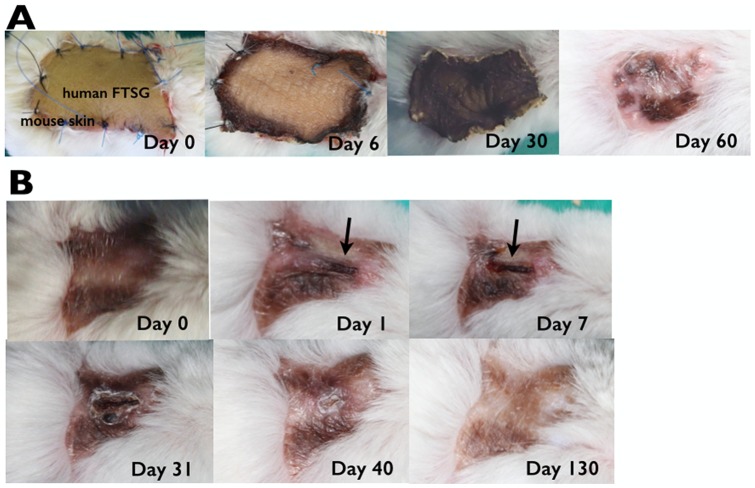
Photographic evolution of the human skin graft in mice. **A, human full-thickness skin graft evolution from the control group.** (a) Day of surgery. (b) Day 6 after surgery. (c) Day 30 after surgery. (d) Day 60 after surgery. Macroscopically, the graft was soft and pliable, resembling normal human skin. Note that the stable human full-thickness skin graft from the control groups took successfully. B, human full-thickness skin graft evolution after placing the compression device for three cycles. (a) Day before the compression device was applied. (b) Day 1 after the three compression cycles. The human skin graft remained folded with a hemorrhagic area in the center of the fold (see arrow). (c) Day 7 after compression cycles. Irreversible damage characterizing a PU can be observed. The center of the fold was occupied by a necrotic and hemorrhagic area (see arrow). (d) Day 31 after the compression cycles. Crusty remnants were observed. (e) Day 40 after compression cycles. Only a small central crusted island could be observed. (f) Day 130 after compression cycles. Complete regeneration of the graft was noticed.

**Figure 3:**
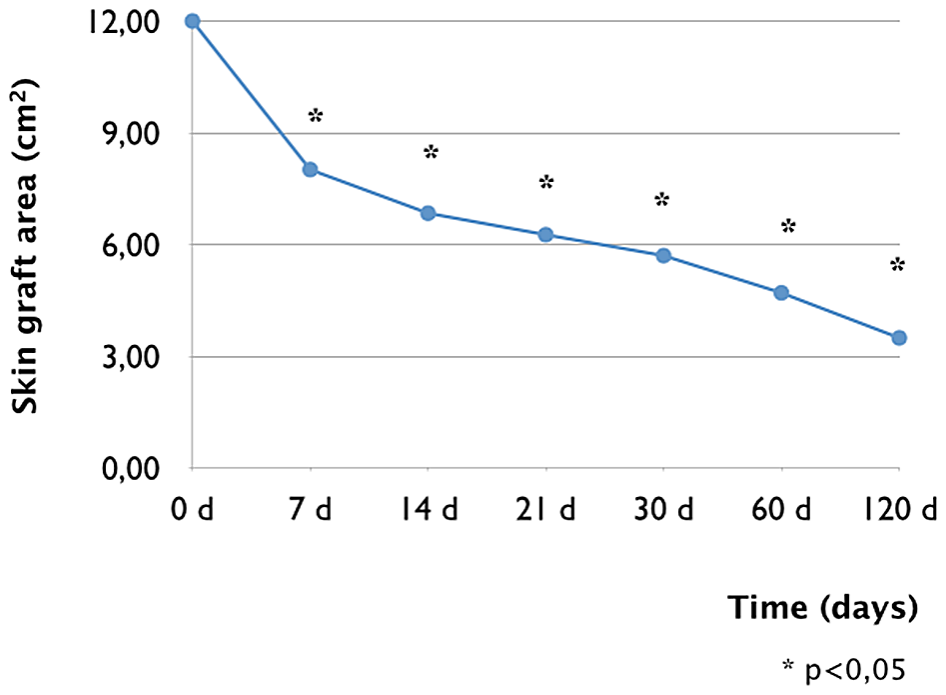
Area in cm^2^ of the human full thickness skin graft on the back of the mice during the first 120 days after surgery. A significant reduction of the skin graft can be observed during the first week after the transplantation.

Stable human FTSGs from the control groups took successfully, as demonstrated by photographic assessment (Fig. 2A). Macroscopically, the graft was soft and pliable after 60 days, resembling normal human skin (Fig. 1A–d). Human FTSGs showed uniform behavior in all studied animals. The FTSGs featured normal coloration (Fig. 2A–a) during the first few days but successively transformed into crusty skin over the first 30 days (Figs. 2A–b and 2A–c). This crust progressively disappeared and was replaced by a fully re-epithelialized area resembling normal and fine human skin after 60 days (Fig. 2A–d). Morphometrical analysis showed the effect of the contraction and retraction of the grafted skin. An important retraction of 33% of the graft was observed in the first 7 days. A progressive decrease with a retraction of 71% after 120 days was reached (Fig. 3).

**Figure 4:**
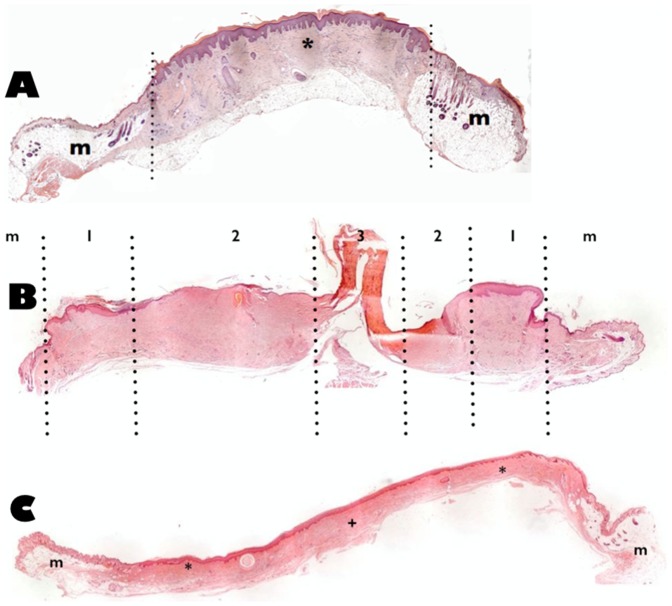
Histological analysis (H&E at 5x, panoramic view) of the mouse and human skin. A, human skin taken 60 days after placement of the human full-thickness skin graft. A clear delimitation between the normal human skin (*) and the mouse skin (m) can be observed. The transition between human and mouse skin has been marked with dotted lines. B, pressure ulcer over the human full-thickness skin graft. 7 days post-compression cycles. Pressure ulcer tissue can be observed as a consequence of mechanical damage. Four zones can be differentiated: m =  receptor mouse skin; zone 1 =  normal human skin; zone 2 =  medium damage human skin; zone 3 =  maximal damage human skin. C, 130 days post-compression cycles. A central area with a stratified epithelium over a non-papillary neodermis (+), a homogeneous and uniform human skin (*) and receptor mouse skin tissue (m) can be observed.

In the histological analysis, a panoramic view showed (Fig. 4A) a clear delimitation between the human skin and the mouse skin. The presence of well-preserved human skin could be observed in the center of the graft which was formed by a stratified epidermis on a dermis with many papillae, resembling human skin. At higher magnification, keratinized squamous epithelium was observed on a papillae dermis with pressure corpuscles. A lymphocytic infiltrate was randomly distributed without specific accumulations. Human dermis (superficial and deep level) was well vascularized, which implies graft stability. The human skin was surrounded by a discrete and well-vascularized dermal layer from the receptor tissue (mouse). The host tissue presented the usual features of mouse skin. This animal model had a fine skin, two or three layers of keratinocytes, a papillary dermis with well-preserved hair follicles and a deep dermis with great development of adipose tissue.

**Figure 5:**
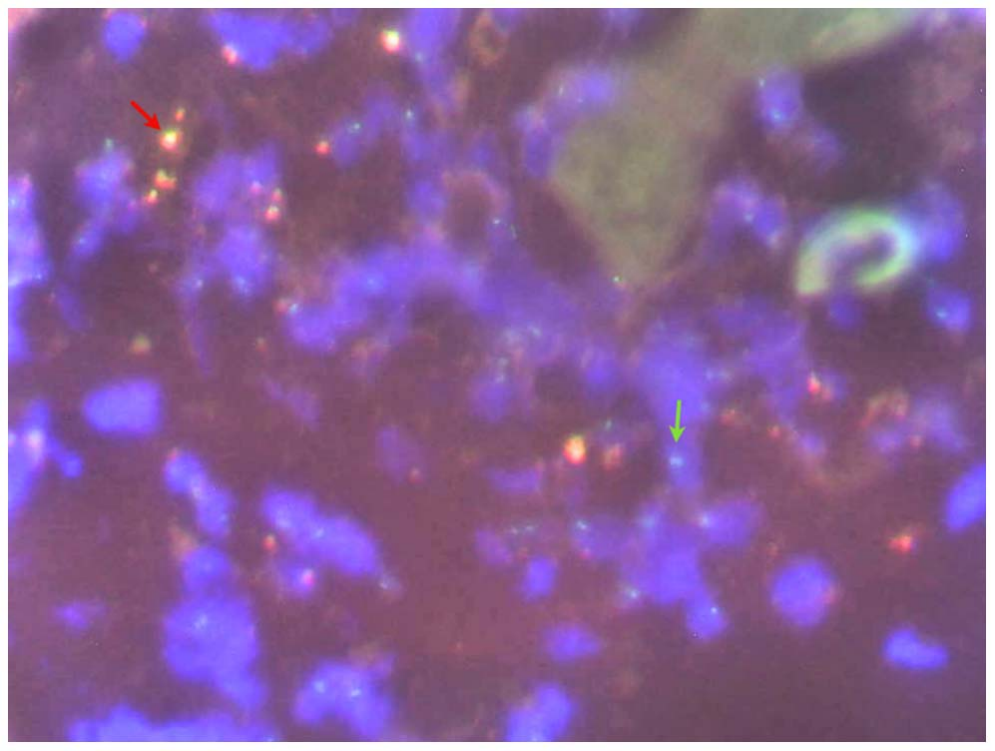
Fluorescence in situ hybridization (FISH) for chromosomes XX and XY. Chromosomes XX from the female human skin and chromosome XY from the male mouse skin were found as green-green dots (X-X, see green arrow) and green-red dots (X-Y, see red arrow). Most cells showing the Y chromosome (red dot) appeared to correspond to mouse cells infiltrating the human tissue.

The human tissue over the mouse receptors was assessed using the FISH technique (Fig. 5). Chromosomes XX from the human skin and chromosomes XY from the mouse skin were found as green-green dots (X-X) and green-red dots (X-Y). Most cells showing the Y chromosome (red dot) appeared to correspond to mouse cells infiltrating the human tissue.

### Pressure ulcers

Placing the compression device on the human skin graft for three cycles induced irreversible damage, characterizing a PU (Fig. 2B). A dermoepidermal necrotic fold covering the entire longitudinal extension under the site of the compression device was observed day 1 post-cycle (Fig. 2B–b). The ischemia produced over the next few days continued to bleed on day 7 post-cycle (Fig. 2B–c). Then, a further evolution towards a retracted and elevated lesion with crusty edges with underlying granulation tissue was observed (Fig. 2B–d). The appearance of the lesion changed completely on day 40 due to the loss of the crust and the formation of a sclerotic surface (Fig. 2B–e). Fully recovered dermoepidermal tissue then emerged. The stability of the human skin graft even after recovery of the PU was striking; the human skin had completely recovered after 130 days (Figs. 2B–a vs. 2B–f).

Histological analysis of the injured human skin was performed systematically in mice at various intervals (days 5, 25, 45 and 130 days post-cycle) after the three ischemic periods of 8 hours. Tissue ischemia was observed during the first days after the cycles of compression as a consequence of mechanical damage (Fig. 4B), reminiscent of PU tissue. The effect of the compression device induced potent tissue degeneration, necrosis in the center and full thickness skin loss involving subcutaneous tissue damage (stage III pressure ulcer). Tissue with a "U" shape was formed by dermal collagen residues. The edges of the tissue were also affected by loss of epithelialization, vascularization and cell population (Fig. 4B, zone 3). These edges were followed by skin areas showing denuded surface, epithelial desquamation and small surface areas covered with crust. The superficial dermis was composed of thick bundles of acellular collagen, while the deep dermis showed signs of cell habitability (Fig. 4B, zone 2). Compression effects were not observed in remote areas (Fig. 4B, zone 1). Finally, this area was bordered by the recipient mouse tissue (Fig. 4B, zone m). The evolution of this tissue damage was studied. Total tissue recovery was observed after 130 days. Fig. 4C shows a sagittal section of the human FTSG. The repair capacity of the human skin could be assessed; zones 1 and 2 of the previous figure (Fig. 4B) were replaced by a homogeneous and uniform human skin, showing the characteristics expected for a reparative epithelium after a significant injury. The central area of Figure 4C (zone 3 in Fig. 4B) showed a stratified epithelium over a non-papillary dermis, which is characteristic of neodermis.

## Discussion

### Human skin grafts

In our initial experiments, human FTSG rejection was observed in all mice using semiathymic and athymic nude mice (unpublished results). Although several papers have reported human skin transplantation to the nude mice, [20]–[24] hypertrophic scarring of the tissue developed instead of viable normal human skin. Yang et al. [24] postulated that although nude mice are immunologically defective of T cells, there might be other immune mechanisms involved, most likely related to the strong antigenicity of the skin, remnant T cells in nude mice (extrathymic lymphocyte populations) and the enhanced role of macrophages and natural killer cells. In the same way, Lin et al. [25] published that NK cells and macrophages could be activated in the absence of T cells or xenoantibodies to directly reject xenografts. To solve this problem, male NOD.CB17-Prkdscid/NCrHsd mice were used in our model. No graft rejection was observed and a viable normal human skin was achieved after 60 days.

Other models of human skin transplantation on mice were used in order to study the rejection process. Murray et al. [26], [27] transplanted 7×7 mm skin graft on a C.B-17 SCID mice. Changes that resembled skin rejection in humans were observed after 2 weeks. Waldron-Lynch et al. [28] used neonatal NOD/SCID/IL2Rc cnull mice, reconstituted with human CD34+ hematopoietic stem cells. A murine skin transplant model in humanized mice was used to test human monoclonal antibody therapy. Racki et al. [29] studied human skin transplantation on immunodeficient mice and rejection following engraftment of allogeneic peripheral blood mononuclear cells. They transplanted 1.5 cm^2^ human skin graft onto NOD-scid IL2rγ^null^ and CB17-scid bg for this purpose. Although all these models are useful for studying the immunology system and the rejection process, non of them provide enough dimensions of normal human skin as our model. Even when a fully new re-epithelialized area resembling normal and fine human skin was available after 60 days, the graft dimensions were about 5 cm^2^. Although it is a 60% less than the initial dimensions (12 cm^2^), this area should be enough to study the PU process or other possible skin damage.

Other models have used genetically engineered human skin on the backs of NOD/SCID mice [30]–[32]. Selected keratinocytes were assembled in a live fibroblast-containing fibrin dermal matrix orthotopically grafted onto mice. Although the authors presented stable human bilayer skin, we could not apply this to our model because human dermis is a more complex structure with a key role in PU generation and resolution.

Our model is based on a four-month stable human FTSG with complete dermal and epidermal layers. Studies have focused on the progress and shrinking of the graft and on the re-epithelialization after the generation of a PU. The graft has shown a high rate of shrinkage in the implant site, specially during the first 7 days. This process, which is known as primary contraction, is the immediate recoil of freshly harvested grafts as a result of the elastin in the dermis. The more dermis the graft has, the more primary the contraction that will be experienced [33]. In our model, more than half of the original size of the graft was observed after the FTSG reached a stable phase. The maximum dimensions of the back of the mice (4×3 cm) were used to achieve enough human graft after 60 days to develop the PU. The evolution of the graft was towards the initial formation of a crust related to the dermal and epidermal surface area of the graft. After 60 days, the FTSG site was denuded of a crusty layer and a new intensely keratinized and vascularized human stratified epithelium was formed.

Therefore, based on these results, we have used this experimental model to induce PUs in human skin. Skin stability was observed up to 190 days after the FTSG surgery, which demonstrates the effectiveness of our experimental model.

### Pressure ulcers

PU models have been developed using mouse skin [17]–[19], [34], [35]. Our model presents a true human skin pressure ulcer model for the first time (see Table 1).

**Table 1:** Characteristics of the different published pressure ulcer models.

Model	Animal	Compression device	Cycles	Tissue
*Stadler et al*. (2004){Stadler:2004jx}	BALB/c mouse	magnetic plates	12 h compression - 12 h release	mouse skin
*Reid et al*. (2004){Reid:2004ee}	C57BL/6J mouse	magnetic plates	1, 2 or 5 cycles/day of 0.5 h	mouse skin
*Wassermann et al*. (2009){Wassermann:2009ba}	Balb/c nu/nu nude mouse	neodymium magnet	2 h compression - 1 h release	mouse skin
*Garza-Rodea et al*. (2011){delaGarzaRodea:2011 kq}	NOD-LtSz-scid/scid/J mouse	magnetic disks	Only one cycle (4/8/12/14/20 h of clamping)	mouse skin
*Maldonado et al.*	NOD.CB17-Prkdscid/NCrHsd mouse	forceps	8 h compression - 16 h release	human skin

Based on the PU model presented by Stadler et al. [17], we used an original modification, replacing magnets with a mechanical compression device delivering a known, constant and controlled pressure. A modification of the magnet model was published by Wassermann et al. [18] A steel disk was implanted under the gluteus maximus muscle and pressure cycles were applied in conjunction with a magnet. This method could potentially damage the tissue underlying the implanted steel disk and, in particular, the area where the human FTSG was placed in our model. Our model differs from other models because the pressure exerted by the magnets depends on the thickness of the fold and on the position of the magnet. Moreover, we found it more reproducible to put forceps in the same position after every cycle than magnetic disks. After 3 cycles of 8 hours of clamping, irreversible tissue ischemia led to tissue necrosis, which was visible a week after the last cycle. This tissue damage was observed as an ulcer on the edges of the compression device and in the underlying deep dermal and subcutaneous tissue (stage III pressure ulcer).

Ulceration and bleeding could be observed for the rest of the first month. In all cases, recovery of the tissue continuity was evident after 40 days, which corroborated the notion that our model of ischemia had been successful in obtaining an experimental transient PU over human skin. Regenerated epidermis was characterized as a keratinized epidermis without melanocytes and with no papillary dermis, as in human skin defects. The underlying dermis demonstrated parallel bundles of mature collagen with new vascular components. Lymphocytic infiltrates were limited to the interface between the host (mouse) and the grafted (human) tissue.

This study has two main limitations. First, mice immunosuppression does not allow a normal inflammation process. However, as mentioned above, human PUs may be not only a pressure problem [14]. Systemic factors as diabetes, may produce decreased tissue perfusion, poor wound healing, slower epithelialization and immunosuppression [36]. We think that previous models [17]–[19], [34] using completely healthy mice are distant from a clinical setting. Our immunosuppressed model would be more similar to the overall condition that many patients with PUs have: delayed wound healing, altered healing environment, etc. Moreover, from a histological point of view, our model would be more analogous to human PUs than previous models. Nevertheless, further improvements of the model remain to be performed. The second limitation involves the damaged adipose tissue under the human skin. Adipocyte cells come from the mouse, not from the human graft. Although that could be a limitation, we think that it should not affect the cicatrization process as the dermis and epidermis play the main role.

We believe this PU model could represent the most similar situation to human PUs at this time. Further research into any kinds of cellular (i.e. stem cells) or molecular therapies (i.e. growth factors) could be tested directly on damaged human skin after pressure without the ethical issues involved with research in humans. As we described above, the cicatrization time of this model is well-known. Improving this process on our model with new therapies, could potentially mean a direct application in the healing of human PUs.

## Conclusions

To our knowledge, this is the first model where PU has been developed over human skin. In comparison with other mouse skin PU models, future therapies applied in our human skin model could be more realistically extrapolated to a human PU. We think it opens up prospects for testing different cellular or molecular therapies directly over human skin.
